# Merkel cell polyomavirus and its etiological relationship with skin tumors^☆^

**DOI:** 10.1016/j.abd.2023.04.004

**Published:** 2023-07-03

**Authors:** Thiago Rubim Bellott, Flávio Barbosa Luz, Anna Karoline Fausto da Silva, Rafael Brandão Varella, Mayra Carrijo Rochael, Luciana Pantaleão

**Affiliations:** a Department of Pathology, Hospital Universitário Antônio Pedro, Universidade Federal Fluminense, Niterói, RJ, Brazil; b Department of Dermatology, Hospital Universitário Antônio Pedro, Universidade Federal Fluminense, Niterói, RJ, Brazil; c Department of Microbiology and Parasitology, Instituto Biomédico, Universidade Federal Fluminense, Niterói, RJ, Brazil

**Keywords:** ERG transcriptional regulator, Merkel cell carcinoma, Merkel cell polyomavirus, Polyomavirus, Skin neoplasms

## Abstract

Viruses have been frequently identified in several human neoplasms, but the etiological role of these viruses in some tumors is still a matter of controversy. Polyomaviruses stand out among the main viruses with oncogenic capacity, specifically the Merkel cell polyomavirus (MCPyV). Recent revisions in the taxonomy of polyomaviruses have divided the *Polyomaviridae* family into six genera, including 117 species, with a total of 14 currently known human-infecting species. Although the oncogenicity of polyomaviruses has been widely reported in the literature since 1950, the first description of a polyomavirus as an etiological agent of a neoplasm in humans was made only in 2008 with the description of MCPyV, present in approximately 80% of cases of Merkel cell carcinoma (MCC), with the integration of its genome to that of the tumor cells and tumor-specific mutations, and it is considered the etiological agent of this neoplasm since then. MCPyV has also been detected in keratinocyte carcinomas, such as basal cell carcinoma and squamous cell carcinoma of the skin in individuals with and without immunosuppression. Data on the occurrence of oncogenic viruses potentially involved in oncogenesis, which cause persistence and tissue injury, related to the Merkel cell polyomavirus are still scarce, and the hypothesis that the Merkel cell polyomavirus may play a relevant role in the genesis of other cutaneous carcinomas in addition to MCC remains debatable. Therefore, the present study proposes to explore the current knowledge about the presence of MCPyV in keratinocyte carcinomas.

## Introduction

It is estimated that approximately 16% of all cases of cancer worldwide are related to infectious agents, with approximately 10%‒12% of these cases being associated with viruses.^1^, ^2^, ^3^ Human oncoviruses, identified as etiological agents of some types of cancer and with unequivocal oncogenic capacity, currently widely accepted by the scientific community, comprise the human papillomavirus (HPV); hepatitis B virus; hepatitis C virus; Epstein-Barr virus; human herpesvirus 8; human T-cell lymphotropic virus (HTLV); and Merkel cell polyomavirus (MCPyV).^4^, ^5^ This conclusion is based on knowledge accumulated from a large number of experimental, clinical, and epidemiological studies over the past five decades.^1^, ^2^, ^3^, ^4^, ^5^ Although many animal and human diseases caused by viruses can transform normal cells into oncogenic ones, there are few of these diseases consistently associated with neoplasms in humans. Among the viruses that cause persistent infection and have shown oncogenic potential, the polyomavirus (PyV) stands out since it is often found in neoplastic lesions.

It is also noteworthy that due to the ubiquity and persistence of these viruses, their causal association with these neoplasms and other varied non-neoplastic lesions is difficult to interpret. It is postulated that the reactivation and chronicity of these infections may be related to the malignancy process due to chronic tissue injury. Moreover, the concomitant presence of these viral agents has been reported in some tumors, indicating a possible co-stimulatory role of the infectious process.^1^

Oncogenesis is a multifactorial pathway and viral systems support the conception that tumor development occurs through the accumulation of multiple events, whereas the presence of viral DNA in tumor cells is not necessarily a causal factor. Cell transformation caused by a viral infection can lead to activation of oncogenes or inactivation of tumor suppressor genes, making these mechanisms important in viral oncogenesis.^6^, ^7^ The production of oncogenic proteins is the main oncogenic mechanism of some retroviruses, while the inactivation of tumor suppressor proteins occurs in some DNA viruses, such as PyV. For the latter, the inhibition and degradation of regulatory proteins such as the p53 protein and the retinoblastoma protein constitutes their main strategy.^6^, ^7^ Additionally, chronic inflammation, often caused by other infectious agents, can lead to increased cancer risk, as in colon cancer associate with ulcerative colitis. The competence of the immune system is also crucial for infectious and oncogenic control, with immunosuppression being one of the main risk factors for progression to malignancy.^6^, ^7^

Since the discovery of MCPyV in 2008,^8^ the first human PyV unequivocally related to a neoplasm in humans, Merkel cell carcinoma (MCC), a rare neoplasm, possibly of neuroendocrine origin and with an aggressive course, the detection of its viral genome began to be investigated not only in cases of CCM but also in several other skin neoplasms. These include non-melanoma skin cancer (NMSC) or keratinocyte carcinoma (KC), such as basal cell carcinoma (BCC) and cutaneous squamous cell carcinoma (SCC), which aroused great interest in several researchers.^9^, ^10^, ^11^, ^12^, ^13^

## History of polyomavirus and Merkel cell polyomavirus

**Figure 1:**
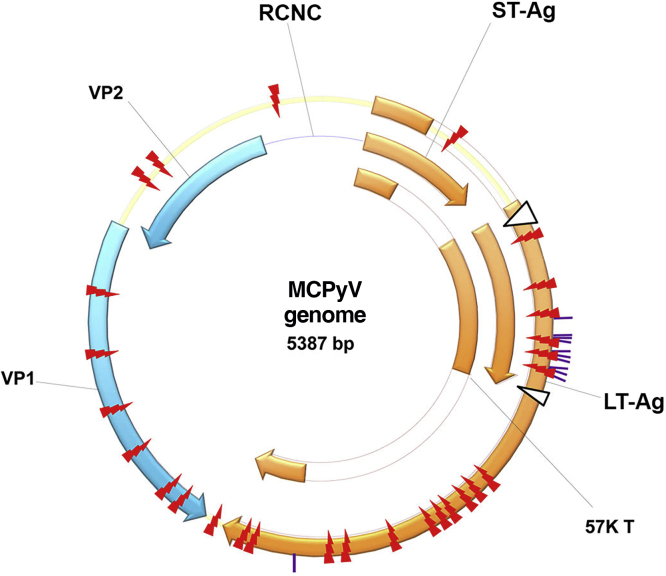
MCPyV genome: non-coding control region (NCCR); the early region, which encodes the large T-antigen (LT-Ag) and small T-antigen (ST-Ag); and the late region. LT-Ag, large T-antigen; ST-Ag, small-T antigen; NCCR, non-coding control region; VP1 and VP2, viral capsid proteins 1 and 2. Source reprinted from: Liu W, et al. 2016.^15^

The first PyV was isolated in 1953 by Ludwik Gross and received this name because it is the causal agent of multiple tumors in rodents (from the Greek *poly*: multiple; and *oma*; tumors). Since then, new species of PyV have been discovered which infect mammals, birds and fish.^14^ These are non-enveloped, icosahedral viruses, approximately 45 nm in size, with double-stranded DNA. Its viral genome is divided into three main regions: the non-coding control region (NCCR), responsible for the elements regulating viral replication and transcription; the early region, which encodes the large T antigen (LT-Ag), and the small T antigen (sT-Ag); and the late region, which encodes the structural proteins expressed after the beginning of DNA replication: viral capsid protein 1 or VP1, viral capsid protein 2 or VP2, and viral capsid protein 3 or VP3, which form the viral capsid (Fig. 1).^15^

**Figure 2:**
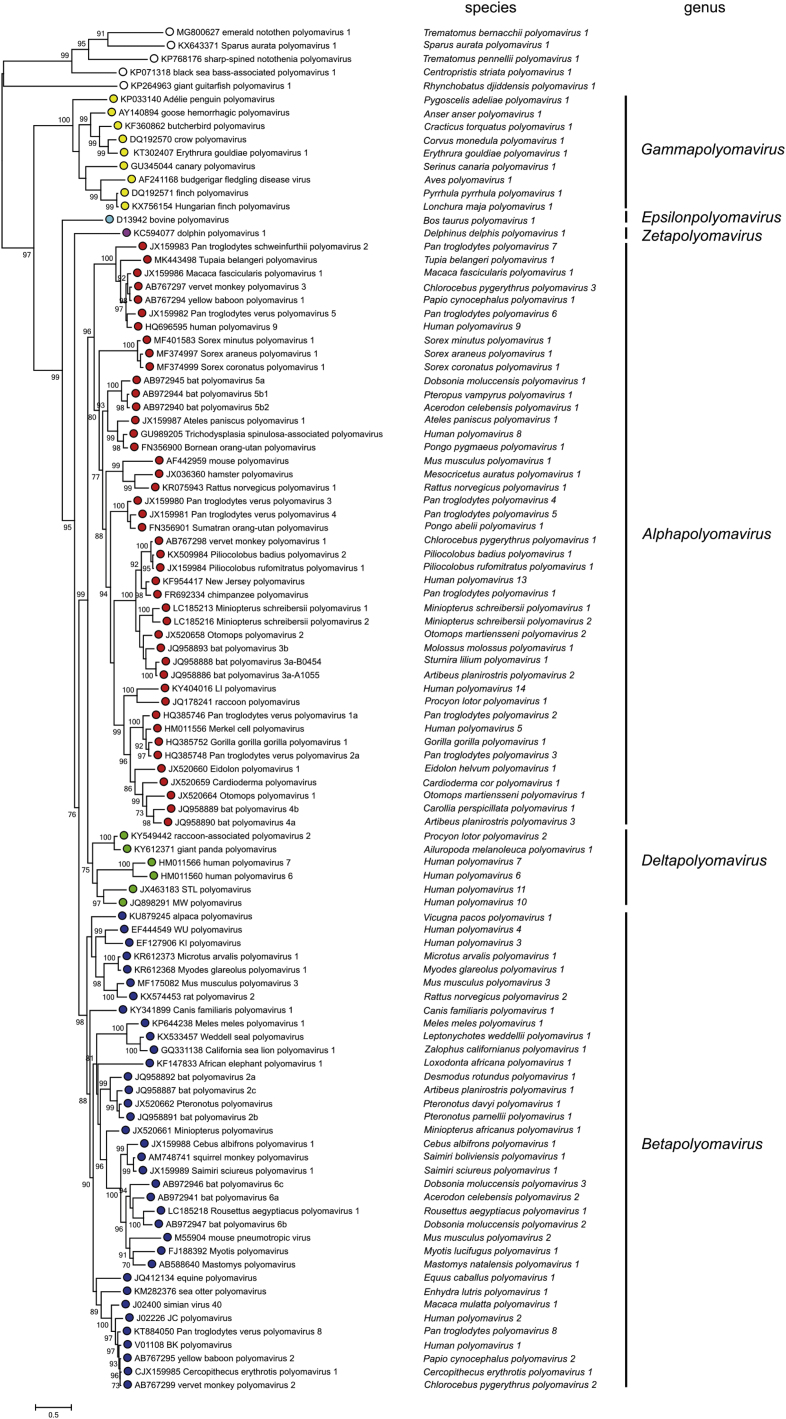
Reviews of PyV taxonomy by the International Committee on Taxonomy of Viruses (ICTV), including more than 100 species and dividing the *Polyomaviridae* family into six genera, plus a group of species without a defined genus: *Alphapolyomavirus*, *Betapolyomavirus*, *Gammapolyomavirus*, *Deltapolyomavirus*, *Epsilonpolyomavirus* and *Zetapolyomavirus*. Source: Moens U, et al.^16^

The pace at which PyV are being discovered has increased enormously over the past 10 years, with constant updates and reviews in their taxonomy performed by the International Committee on Taxonomy of Viruses (ICTV).^14^ Currently, the *Polyomaviridae* family is divided into 6 genera and 117 species, based on the phylogenetic relationships of the LT-Ag amino acid sequence: *Alphapolyomavirus*, *Betapolyomavirus*, *Gammapolyomavirus*, *Deltapolyomavirus*, *Epsilonpolyomavirus* and *Zetapolyomavirus*, plus a group of species still without a defined genus.^16^ The genus *Alphapolyomavirus* comprises PyV species that infect humans and animals, including the human polyomavirus (HPyV) 5 or MCPyV, HPyV8 or polyomavirus associated with trichodysplasia spinulosa (TSPyV), HPyV9, HPyV12 and HPyV13; the genus *Betapolyomavirus* includes species that infect humans and animals, including HPyV1 and HpyV4; the genus *Deltapolyomavirus* contains HPyV6, HPyV7, HPyV10 and HPyV11; the genus *Gammapolyomavirus*, and *Epsilonpolyomavirus* and *Zetapolyomavirus*, described recently, have other species that infect mammals and fish, in addition to the species of PyVs without a defined genus (Fig. 2).^14^, ^16^, ^17^

**Figure 3:**
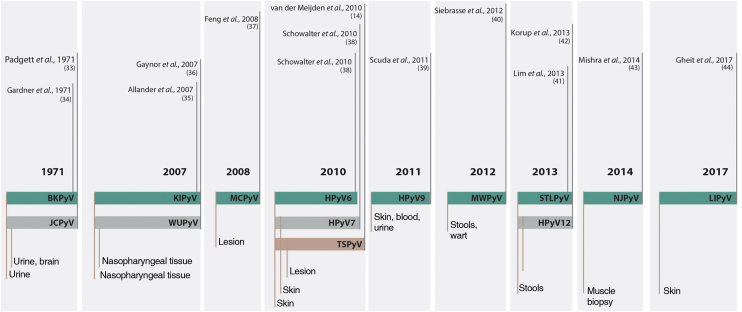
Chronology of fourteen human-infecting PyV species discovered since 1971. Source: adapted from Prado J, et al. 2018.^18^

Up to 2007, only two species had been described as members of the family that infected humans, the HPyV1 or BK polyomavirus (BKPyV); and the HPyV2 or John Cunningham polyomavirus or JC Virus (JCPyV). From this date on, due to the evolution of technology for genetic sequencing, another 12 HPyVs were described and named according to the chronological order of their discovery, totaling the 14 currently known species that infect humans (Fig. 3).^16^, ^17^, ^18^

Several of these species have an affinity for cutaneous tissue, including HPyV6, HPyV7, HPyV9, TSPyV, and MCPyV.^19^, ^20^, ^21^, ^22^ Important diseases caused by PyV are known to occur in immunosuppressed patients, such as progressive multifocal leukoencephalopathy in patients with acquired immunodeficiency syndrome by JCPyV, hemorrhagic cystitis in patients after allogeneic hematopoietic stem cell transplantation by BKPyV, among others. About 10% of kidney transplant patients develop BKPyV-associated nephropathy, with loss of graft function in up to 50% of the cases.^23^

The classification created by the International Agency for Research on Cancer (IARC) of the World Health Organization (WHO) divides carcinogenic agents into groups, separated by their greater or lesser carcinogenic action in humans, based on evidence: Group 1 as carcinogenic agents in humans; Group 2A as likely human carcinogens; Group 2B as possibly human carcinogens; Group 3 as agents unrelated to carcinogenesis in humans. Among the viral agents involved or potentially co-participants in oncogenesis are HPyV, notably MCPyV. Based on this WHO classification, MCPyV has been classified as Group 2A.^5^, ^24^, ^25^

Serological evidence supports the persistence of HPyV infection. By measuring antibodies against the viral capsid protein VP1, it can be inferred that exposure to HPyV occurs in childhood, with an increase in seroprevalence over the years, and measurement of very low levels up to 18 months of life and close to 80% by 5 years of age.^8^ Serological assays performed specifically for MCPyV also confirm this viral ubiquity, where 80% of the adults had antibodies against the virus.^26^

The presence of MCPyV DNA, showing variable viral loads by the PCR technique, has also been described in several non-tumor tissues, such as skin, peripheral blood, gastrointestinal, respiratory, and urinary tracts, adrenal gland, spleen, bone marrow, stomach, gallbladder, pancreas, heart, and aorta, albeit with a relatively low viral load, between 0.00026 and 0.22 copies per cell.^27^, ^28^, ^29^, ^30^ On average, the number of copies of the MCPyV viral genome was 60 times lower in healthy tissues, when compared to MCC samples.^28^

Another factor supporting the fact that MCPyV infection is ubiquitous and asymptomatic is that 60% to 80% of the population continuously shed MCPyV through healthy skin.^31^ Recent reports also report that fibroblasts present in the dermis can support viral replication and can be genuine host cells for this virus.^32^

## Merkel cell carcinoma and Merkel cell polyomavirus

Although the oncogenicity of PyV has been widely reported in the literature since 1950, the first description of PyV as an etiological agent of neoplasms in humans was only made in 2008 with the description of MCPyV, present in approximately 80% of cases of MCC and implicated as the etiological agent of this tumor.^8^

**Figure 4:**
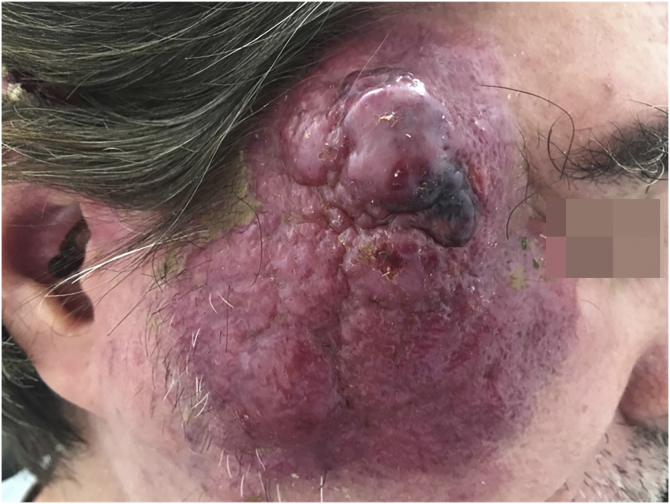
A case of Merkel cell carcinoma in an immunosuppressed patient with acquired immunodeficiency syndrome.

MCC is a rare neoplasm, possibly of neuroendocrine origin, with an aggressive course. This tumor usually affects immunosuppressed patients of advanced age, in places of chronic photo exposure. The lesion usually presents as a rapidly-growing erythematous papule or nodule without associated symptoms. Most of these lesions have three or more characteristics summarized in the acronym AEIOU: A for “Asymptomatic nodule”; E for “Expanding rapidly”; I for “Immunosuppressed host”; O for “Older than age 50”; and U for “UV-exposed sites or fair skin”^33^ (Fig. 4).

**Figure 5:**
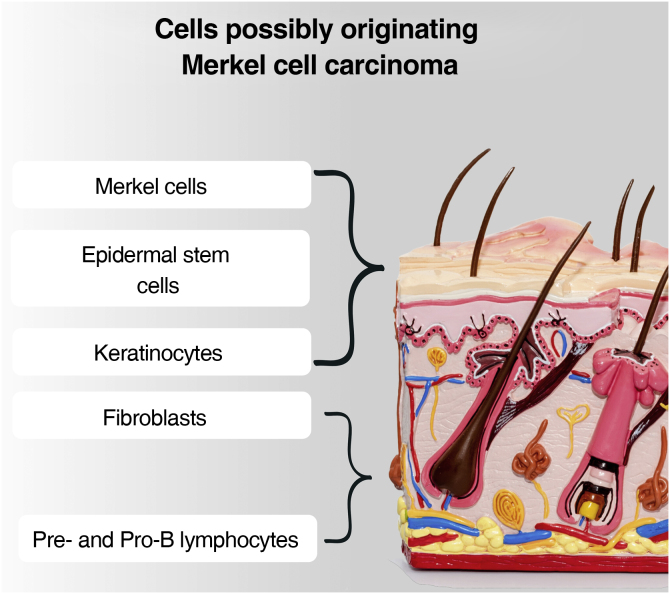
Main cells postulated as responsible for the genesis of MCC

Although the tumor cells share morphological and histological characteristics with Merkel cells, there is a debate about the origin of this tumor. This is due to the fact that Merkel cells are post-mitotic, being in the terminal differentiation stage and not performing mitosis, making the origin of a tumor very difficult. Another question is that MCC rarely affects sites where Merkel cells are present, such as the palms, soles, nail bed, and oral and genital mucosa. Added to this fact: the absence of descriptions of dysplastic lesions or precursors of this tumor; the location of Merkel cells in the epidermis, while the MCC is located in the dermis; the difference in phenotypes between the Merkel cell and the MCC. Therefore, it is postulated that other cells may be responsible for the origin of this tumor, and possible candidates include epidermal stem cells, keratinocytes (the predominant cells in all epidermal cell layers), dermal fibroblasts, pro-B or pre-B cells (Fig. 5).^4^, ^33^, ^34^

In this rare cutaneous neoplasm, the viral DNA is clonally integrated into the host cells DNA as a result of a mutation. The pattern of clonal integration of the MCPyV DNA into the host cells DNA suggests that the viral infection occurred prior to the clonal expansion of MCC neoplastic cells.^15^ In addition to this mutation that caused clonal integration, a second mutation is required for the development of this neoplasm, which partly explains the rarity of this entity. The second mutation can be induced by exposure to UV rays and is represented by truncation of the MCPyV LT-Ag. This mutation will ultimately lead to inhibition of the RB tumor suppressor protein in these infected cells, and an increase in E2F transcription factors, triggering the S phase of the cell cycle, similar to what occurs in the oncogenesis mechanism by the HPV E7 oncoprotein. The LT-Ag also contributes to oncogenesis by stimulating angiogenesis and telomerase activity, in addition to inhibiting intracellular pathways that lead to apoptosis. The ST-Ag also seems to have multiple oncogenic functions and would act in conjunction with LT-Ag to complete this process.^35^ Feng et al.^8^ describe the integration of the MCPyV genome into the cell genome prior to the clonal expansion of neoplastic cells, in addition to these tumor-specific mutations of LT-Ag truncation.^8^, ^36^ These mutations have also been reported in a number of cases of chronic lymphocytic leukemia^37^ and non-small cell lung cancer.^38^ The viral genome has also been found in squamous cell cancer and cervical adenocarcinomas,^39^ and lymphoid leukemias,^40^, ^41^ although there is still no etiological proof of MCPyV in malignancies other than MCC.

Recent studies have given greater prominence to the oncogenic role of ST-Ag than previously thought, and a key difference between MCPyV and other PyVs is that the ST-Ag seems to be directly involved in MCPyV-induced neoplastic transformation in MCC.^42^

Liu et al.,^32^, ^42^ after examining epidermal keratinocytes, dermal fibroblasts, Merkel cells, and many other cell types in the skin, found that only dermal fibroblasts allow MCPyV entry, transcription, and replication.^32^, ^42^ These fibroblasts, when treated with epidermal growth factor and basic fibroblast growth factor, were particularly effective in supporting MCPyV infection, and as these growth factors are typically upregulated at skin wound sites, it is suggested that the healing process can induce a tissue microenvironment favorable for MCPyV infection and replication.^32^, ^42^

It has been suggested, in studies initially carried out in the Australian population, that the participation of MCPyV in the genesis of MCC is uncommon in tumors located in photodamaged areas of fair-skinned individuals.^43^ This information is corroborated by the lower frequency of detection of viral DNA in cases of MCC associated with squamous differentiation, which usually occurs only in photodamaged areas, leading to the hypothesis that there is an alternative pathway of oncogenesis in these tumors, independent from the virus.^44^, ^45^

The rate of viral positivity seen in tumors in regions with and without photo exposure was also studied by Dabner et al.,^46^ using solar elastosis in the dermis and epidermal dysplasia as histopathological parameters of chronic actinic damage. The results demonstrate the same pattern, with a lower viral presence in tumors in photo-exposed areas. In agreement with this, Paik et al.^45^ also demonstrated a lower positivity for MCPyV in CCM by immunohistochemistry in Australian individuals, with a detection rate of 7.7% (3 in 39) in tumors located on the head and neck and of 24.6% (16 in 65) in other body locations. These studies reinforce the possibility that tumors located in photo-exposed regions, such as the head and neck, have other carcinogenesis pathways unrelated to viral presence, which would explain the low detection by this method in these tumors.^45^

There are, however, studies that point in the opposite direction, demonstrating a greater presence of viral DNA through the PCR technique in NMSC of photoexposed sites. This result raises the hypothesis that viral carcinogenesis acts as a complementary factor to UV-induced carcinogenesis.^47^

To date, there is yet no gold standard test for the detection of MCPyV, and even the combined use of PCR and immunohistochemistry (IHC) shows suboptimal results.^48^ Previous studies that used the two combined techniques for the detection of MCPyV in MCC had a higher positivity with the PCR technique suggesting it is more sensitive.^44^, ^49^, ^50^, ^51^, ^52^ However, other authors have described the detection of MCPyV in MCC with these combined techniques presenting a greater viral detection with the IHC technique. They attributed this finding to the great diversity of methodologies in the PCR technique for MCPyV, such as the use of a primer with different genetic sequences; a number of primer sets of the detected virus; differences in the human reference sequence that is amplified; and the cutoffs used to consider a test positive. It is possible that the most sensitive and specific PCR assay for detecting MCPyV has yet to be compared with IHC assays.^48^

## Merkel cell polyomavirus-related non-melanoma skin cancer and keratinocyte carcinomas

Non-melanoma skin cancer (NMSC) or keratinocyte carcinoma (KC) basically encompasses all skin malignancies that are not related to melanoma. However, most of these tumors correspond to basal cell carcinoma (BCC), cutaneous squamous cell carcinoma (SCC) an its variants.^53^ KC does not include other rarer types of cutaneous malignancies with different behaviors, such as Merkel cell carcinoma itself, dermatofibrosarcoma, cutaneous lymphomas, sebaceous carcinomas, angiosarcomas, and other tumors of cutaneous adnexa.^54^

Considering the role of MCPyV in the development of MCC and its detection in several other tissues and tumors in the human body, several researchers started to investigate a possible etiological role of this virus in the genesis of other tumors, mostly in KC.^55^ In most of these studies,^9^, ^10^, ^11^, ^12^, ^13^ MCPyV DNA was detected using the PCR technique; however, there are some studies in which LT-Ag was also investigated using the IHC technique, most often demonstrating lower positive results than with the PCR technique.^55^

Kassem et al.^10^ demonstrated the presence of MCPyV through the PCR technique in 36 of 96 (37.5%) BCC cases affecting immunocompetent patients. Regarding BCC in immunosuppressed patients, the values were surprisingly high, being positive in 72.2% of the cases. The same authors described the presence of MCPyV in 7 of 28 cases (25%) of SCC in immunocompetent patients. Similar to what happened with BCCs, the percentage rose to 52% in immunosuppressed patients. High positivity has also been described in Bowen's disease, with 17.4% positivity in immunocompetent patients and 69% in immunosuppressed ones.

Also in 2009, Dworkin et al.^9^ described MCPyV positivity in 26 of 177 (15%) SCCs in immunocompetent patients.^9^ In addition to the positivity in SCC, 17% of positivity was also described in the skin adjacent to the tumor (11 of 63 samples). There was, between paired cases (tumor and skin adjacent to the tumor), an agreement of 87% (presence or absence of viral DNA in both specimens). This finding suggests that, despite the absence of specific histopathological changes, the virus could already be present in the perilesional area, leading to the future development of the tumor.

Zur Hausen et al.^56^ evaluated a total of 56 KC in 11 immunosuppressed patients (25 SCC, 13 Bowen's disease, and 18 BCC cases) and 147 tumors in 125 immunocompetent patients (28 SCC, 23 Bowen's disease, and 96 BCC cases).^56^ Detection of MCPyV positivity was significantly more frequent in cutaneous tumors of immunosuppressed patients in the three types of tumor, with statistical significance (p < 0.001). All PCR products were also sequenced in this study, and tumor-specific mutations in the LT-Ag area could not be detected. The viral load was also analyzed in this study, and in the vast majority of KC cases, the MCPyV viral load was well below the MCPyV-positive CCM cases used as controls.^56^

Wieland et al.^57^ analyzed the presence of MCPyV by PCR in 355 skin samples, including a wide range of KC, melanomas, benign lesions, and perilesional healthy skin.^57^ Positivity for the virus was found in three of 21 BCC (14%), one of six SCC (17%), one of four Bowen’s disease (25%), three of seven keratoacanthomas (43%), two of 12 melanomas (17%), no positive cases in viral warts and actinic keratoses, and detection in eight of 34 specimens of healthy perilesional skin (24%). Viral DNA was found in 30 of the 34 MCC cases used as controls, with a viral load higher than that found in KC, a result similar to that described by Zur Hausen et al.^56^, ^57^

Garneski et al.^43^ found a 13% MCPyV positivity rate in SCC cases, where two of the 15 studied SCC lesions harbored viral DNA.^43^ On the other hand, Reisinger et al. ^58^ investigated LT-Ag positivity through immunohistochemistry (IHC), using the CM2B4 antibody in 20 patients with Merkel cell carcinoma and in some BCC and SCC from these same patients. A total of 12 of these patients had BCC and SCC that developed after MCC detection, and the LT-Ag test by IHC using the CM2B4 monoclonal antibody was negative in all 12 cases. Conversely, these patients had a high positivity of 92% (11 of 12) for LT-Ag verified by immunohistochemistry in their Merkel cell carcinomas.^58^ Rollison et al.^59^ described a 38% MCPyV DNA positivity in fresh frozen SCC tissue samples obtained from immunocompetent patients. The presence of MCPyV DNA has been associated with high levels of circulating anti-MCPyV antibodies.^59^ This significant percentage in immunocompetent patients may be due to the fact that the samples were studied immediately after their removal and not after processing the material through paraffin, preventing the possible partial degradation of the genetic material present.

In line with these studies, Mertz et al.^60^ detected MCPyV DNA by PCR in two (10%) of 20 patients with Bowen's disease. In one of these patients, who was immunosuppressed due to a kidney transplant, several other skin lesions were also evaluated, with positivity for MCPyV in four of four viral warts (100%), three of six Bowen's disease lesions (50%), and two of three seborrheic keratosis (66.6%). In a second cohort of immunosuppressed patients submitted to kidney transplantation, MCPyV DNA was found in one of 16 viral warts (6.3%), and in two of 21 Bowen's disease lesions (9.5%). In immunocompetent individuals, MCPyV DNA was found in two of 30 viral warts (6.7%) and two of 24 Bowen's disease lesions (8.3%).^60^

The same researchers,^13^ in 2013, found positivity for the virus in 28 of 71 (39.4%) BCCs removed from immunocompetent patients. This rate rose to 47.1% in immunosuppressed patients, where eight of 17 BCC cases harbored viral DNA. In contrast to the frequent viral detection by PCR in this study, immunohistochemistry to search for LT-Ag in the tumor tissue was positive only in two of the 17 cases of BCC that affected immunosuppressed patients, being negative in all BCCs of immunocompetent patients.^13^ In that same year, Murakami et al.^61^ found four of 30 (13%) SCC positive for MCPyV, and of these four, two were also positive for the presence of LT-Ag by immunohistochemistry. The same study also described total negativity for the presence of MCPyV by PCR in ten cases of BCC, despite the fact that the small total number of cases made the analysis difficult.

Becker et al., after confirming the presence of MCPyV in 45 of 53 (84.9%) patients with MCC, described viral positivity in three of 24 (12.5%) BCC cases.^62^

Neto et al.^62^ also performed a recent analysis of the presence of MCPyV in cutaneous tumors, analyzing ten cases of BCC and ten of SCC. Using the PCR technique with the extraction of genetic material from material stored in paraffin blocks, MCPyV genetic material was detected in four of the 20 cases, in comparison to the presence in 100% of the 20 cases of MCC also analyzed in this study.^63^

Ridd et al.,^63^ after investigating the presence of MCPyV DNA using the PCR technique in 85 cases of SCC, 37 keratoacanthomas, 28 Bowen's disease lesions and 6 actinic keratoses, found positivity in only one case of keratoacanthoma. The PCR technique of this study was validated in an MCC cohort, which showed that 54% (7/13) of the tumors were positive for the presence of MCPyV.^64^

Kaibuchi-Noda et al.^65^ reported a case of an immunosuppressed patient which one year after having an SCC resected, had metastases diagnosed as MCC, with the MCPyV DNA screening performed through the PCR technique and the LT- Ag by the IHC technique.^65^ The study showed that MCPyV DNA was detected in the SCC lesion, indicating that the MCPyV infection occurred at least one year before the occurrence of metastatic MCC. However, the number of MCPyV copies per cell in the MCC lesion was higher than in the SCC lesion, and the LT-Ag screening by IHC was positive only in the MCC, raising doubts about whether the MCPyV actually participated in the genesis of both tumors or only in the MCC.^65^

Recent studies also indicate the presence of other HPyV in keratoacanthomas and other KC. Beckervordersandforth et al.^66^ described the presence of HPyV6 using PCR in several tumors included in the NMSC group, demonstrating that 25 of 59 (42.3%) keratoacanthomas had genetic material, as well as 23 of 109 (21.1%) cases of basal cell carcinoma and eight of 86 (9.3%) cases of squamous cell carcinoma.^66^

The presence of HPyV6, as well as that of other HPyV, such as MCPyV, TSPyV, HPyV6, HPyV7, HPyV9, and HPyV10, has also been investigated in SCC of patients with melanoma receiving BRAF inhibitors.^67^ To date, the only identified factors that contribute to the development of SCC in these patients are mutations in the RAS genes. However, these mutations explain only 60% of the tumors, so it is important to identify what is causing the remaining tumors. Eighteen biopsy specimens from six patients were evaluated, and ten (62%) of the 16 samples contained RAS mutations, with HPyV9, HPyV10, and TSPyV DNA absent in the samples, whereas MCPyV DNA was present in 13 of 18 samples (72.22%), and HPV, HPyV6 and HPyV7 DNA were present in all samples. Overall, the viral load found in this study was low, with the exception of HPyV6, which was also detected using the IHC technique with the VP1 core protein antigens.^67^ Haeggblom et al. described the presence of PyV in keratoacanthomas and Spitz nevi and, although there was no predominance of any specific species, viral genetic material was found by PCR in 20 of 47 lesions.^68^

A study carried out by Toptan et al.^69^ investigated the presence of this virus in hundreds of tumors, including BCC and SCC, using an antibody in the IHC technique that simultaneously detects a wide range of HPyV, including MCPyV. The presence of MCPyV was not detected in these cutaneous carcinomas, and rare cells labeled with this antibody in some sporadic tumors were interpreted as transient and incidental infections.^69^

Studying 96 skin lesions, Bellott et al. ^12^ detected the presence of MCPyV in 31% of these cases, with 23 (33.3%) of 69 cases of basal cell carcinoma, two of 11 SCC (18%), two of four Bowen's disease lesions (50%) and, among the other 11 skin lesions, four (36.3%) were positive for viral DNA (two actinic keratoses, one dermatofibrosarcoma, and one keratoacanthoma). ^12^ A more recent study by the same author,^70^ this time analyzing the presence of the MCPyV viral genome using PCR and the LT-Ag using IHC, in 103 KC samples from 91 patients. A total of 44 (42.7%) tumors were positive for the virus via PCR, 34 of 85 (40%) in the BCC group, six of 12 (50%) in the SCC group, two of four (50%) in Bowen's disease lesions and one of two (50%) in keratoacanthomas. The IHC result show total positivity in 18 cases (17.7%), seven cases of positivity in the tumor only (6.9%), eight in the peritumoral infiltrate (7.9%), and three in both (3%).^70^

Costa et al.^71^ also carried out the study of several HPyV through the PCR technique in cutaneous tissues, including cases of KC, melanoma, Kaposi sarcoma, MCC, and normal skin. MCPyV was the most commonly found HPyV, present in two of 20 (10%) BCC and three of 20 (15%) SCC cases, in addition to not being present in a total of 20 cases of normal skin.^71^

The main studies that investigated the presence of MCPyV viral particles in KC are grouped in chronological order in Table 1.

**Table 1:** Main studies, in chronological order, which investigated the presence of MCPyV viral particles in KC

Authors	Investigated tissues	PCR	Result	IHC	Result
Kassem et al. 2009	BCC, SCC, BD	Yes	BCC IS: 13 of 18 (72.2%)	No	
		*Primers for LT3 and VP1	SCC IS: 13 de 25 (52%)		
			BD IS: 9 of 13 (69%)		
			BCC IC: 36 of 96 (37.5%)		
			SCC IC: 7 of 28 (25%)		
			BD IC: 4 of 23 (17.4%)		
Dworkin et al. 2009	SCC	Yes	SCC IC: 26 of 177 (15%)	No	
		*Primers for LT3 and VP1	Skin adjacent to SCC: 11 of 63 (17%)		
Zur Hausen et al. 2009		Yes	18 BCC IS^1^	No	
		**Primers not described	25 SCC IS^1^		
			13 BD IS^1^		
			BCC IC: 36 of 96 (37.5%)		
			28 SCC IC^1^		
			23 BD IC^1^		
Wieland et al. 2009		Yes	MCC: 30 of 34 (88%)	No	
		*Primers for LT1 and LT3	MCC Mx^2^: 5 of 5 (100%)		
			CBC IC: 3 of 21 (14.2%)		
			SCC IC: 1 of 6 (16.6%)		
			BD IC: 1 of 4 (25%)		
			Keratoacanthoma IC: 3 of 7 (42.8%)		
			Skin adjacent to KC: 8 of 34 (23.5%)		
Garneski et al. 2009		Yes	MCC USA^3^: 11 of 16 (69%)	Yes	
		*Primers for ST-Ag and LT-Ag	MCC AUS^4^: 5 of 21 (24%)		
			SCC: 2 of 15 (13%)		
			Photo-exposed skin: 0 of 15		
Becker et al. 2009		*Primers for LT-Ag	MCC: 45 of 53 (84.9%)	No	
			BCC: 3 of 24 (12.5%)		
Ridd et al. 2009		*Primers for LT1, LT3 and VP1	SCC: 0 of 85	No	
			BD: 0 of 28 Keratoacanthoma: 1 of 37 (2.7%)		
			Actinic keratosis: 0 of 6		
Reisinger et al. 2010		No		Yes	MCC: 15 of 20 (75%)
				*Antibody	BCC^5^: 0 of 9
				Monoclonal	SCC^5^: 0 of 3
Mertz et al. 2010		Yes	BD IS: 2 of 20 (10%) and 2 of 21 (9.5%)^6^	No	
		*Primers for LT1, LT3 and VP1	BD IC: 2 of 24 (8.3%)^6^		
Rollison et al 2012		Yes	SCC: 55 of 145 (38%)	No	
		*Primers for LT-Ag			
Mertz et al. 2013		Yes	BCC IS: 8 of 17 (47.1%)	Yes	BCC IS: 2 of 17 (11.8%)
		*Primers for LT-Ag	SCC IS: 4 of 15 (26.7%)	*Antibody	SCC IS: 1 of 15 (6.7%)
			BCC IC: 28 of 71 (39.4%)	Monoclonal	BCC IC: 0 of 71
			SCC IC: 17 of 60 (28.3%)	CM2B4	SCC IC: 0 of 60
			Normal skin: 10 of 47 (21.3%)		
Murakami et al. 2011		Yes	BCC IC: 0 of 10 (0%)	Yes	SCC IC: 2 of 4^7^
		*Primers for LT3 and VP1	SCC IC: 4 of 30 (13%)	*Antibody	
				Monoclonal	
				CM2B4	
Bellott et al. 2017		Yes	BCC IC: 23 of 69 (33.3 %)	No	
		*Primers for LT3	SCC IC: 2 of 11 (18%)		
			BD IC: 2 of 4 BD (50%)		
			Keratoacanthoma IC: 1 of 1		
Neto et al., 2019		Yes	MCC: 13 of 13 (100%)	No	
		*Primers for LT-Ag	KC: 4 of 20 (20%)		
Costa et al. 2021		Yes	MCC: 13 of 20 (65%)	No	
		*Primers for LT-Ag	BCC: 2 of 20 (10%)		
			SCC: 3 of 20 (15%)		
Bellott et al. 2022		Yes	BCC IC: 34 of 85 (40%)	Yes	IHC T^8^: 18 of 101 (17.8%)
		*Primers for LT3	SCC IC: 6 of 12 (50%)	*Antibody	IHC ST^9^: 10 of 101 (9.9%)
			BD IC: 2 of 4 (50%)	Monoclonal	IHC SI^10^: 8 of 101 (7.9%)
			Keratoacanthoma: 1 of 2 (50%)	CM2B4	

IS, Immunosuppressed; IC, Immunocompetent; 1, Data not available in the article; 2, Mx2 – metastases; 3, United States of America; 4, Australia; 5, BCC and SCC from patients with MCC with positive IHC for MCPyV; 6, two cohorts investigated in the same study; 7, IHC performed only in PCR-positive cases; 8, IHC-T: Total staining, staining of tumor cells and lymphocytic cells in the peri-tumoral infiltrate; 9, IHC-ST: Staining of tumor cells only, in association or not with peri-tumoral lymphocytic infiltrate cells; 10, IHC-SI: Staining only cells from the peritumoral lymphocytic infiltrate.

*When primers were described.

**When primers were not described.

## Discussion

There are several assumptions for the variation in the prevalence of MCPyV in KC among different studies and the divergence of these results from those using IHC.

The detection of MCPyV DNA by the PCR technique can be affected by the primers that were used. In fact, most studies have applied the LT-Ag primers originally used by Feng et al.,^8^ which are considered superior to primers directed against VP1.^55^

The quality of the samples can also affect the outcome of the PCR reaction, as several studies have reported that the detection of MCPyV DNA in fresh frozen tissues is more reliable when compared to detection in paraffin-fixed specimens.^55^

Viral DNA is extracted from a tumor fragment, accessing only a subset of the cells present in the neoplasm. There is a possibility that there are viral particles present in tumor sites that have not been analyzed simultaneously by PCR and IHC. The tumor consists of several heterogeneous tumor cell lineages, immune cell infiltrates such as lymphocytes, endothelial cells, and cancer-associated fibroblasts. Knowing that fibroblasts can support viral replication and can be genuine host cells for this virus,^32^ and also that infiltrating monocytes can be reservoirs for the virus,^72^ positive PCR products can derive from viral infection in these cells.

PCR-based methods are also prone to contamination. It is postulated that MCPyV is chronically shed through the skin and may be part of the skin microbiota,^73^ so contamination during sample collection and handling cannot be excluded.

The simultaneous detection of LT-Ag by IHC and its genetic material by PCR in tumor tissues is evidence of active participation of the virus in the maintenance of cancer growth, and all these studies demonstrate that to help elucidate whether MCPyV is indeed associated with the genesis of KC, further epidemiological studies are still required.^73^

Other steps that are also important for the elucidation of the possible etiological role of MCPyV in tumors other than MCC are:

• The status of the viral genome must be determined, whether episomal or integrated, as the integration of the viral genome seems to be a prerequisite for the development of the neoplastic process associated with this virus^55^;
• The gene encoding LT-Ag must be sequenced to determine whether tumor-specific mutations of truncated LT-Ag are expressed^55^;
• *In situ* hybridization for viral DNA or RNA should be performed to ensure that viral sequences are detected in tumor cells and not in other cell types adjacent to the tumor.^55^

Tumor samples at different stages of development should also be analyzed, as the virus may be involved only early in the neoplastic process and is not required for tumor progression.^55^ In fact, an attack-and-escape mechanism for the oncogenic action of MCPyV in some cases of MCC has been suggested by Houben et al.,^74^ where viral oncogenesis would only be essential to trigger the process.^74^

## Conclusion

Several pieces of information, such as the high seroprevalence of MCPyV in the human population, the *in vitro* oncogenic potential of LT-Ag and ST-Ag, and its etiological role in MCC, suggest that MCPyV may play a role, alone or in association with other factors, in other types of cancer in humans, especially in individuals with compromised immune systems; however, this causal relationship between MCPyV and KC cannot be established yet.^55^

## Financial support

The present study is part of the main author doctoral thesis and was awarded a research grant from FUNADERM (*Fundo de Apoio à Dermatologia*), an entity of the Brazilian Society of Dermatology.

## Authors' contributions

Thiago Rubim Bellott: Design and planning of the study; data collection, or analysis and interpretation of data; drafting and editing of the manuscript or critical review of important intellectual content; collection, analysis, and interpretation of data; effective participation in research orientation; intellectual participation in the propaedeutic and/or therapeutic conduct of the studied cases; critical review of the literature; approval of the final version of the manuscript.

Flávio Barbosa Luz: Design and planning of the study; drafting and editing of the manuscript or critical review of important intellectual content; collection, analysis, and interpretation of data; effective participation in research orientation; intellectual participation in the propaedeutic and/or therapeutic conduct of the studied cases; approval of the final version of the manuscript.

Anna Karolinne Fausto: Effective participation in research orientation; intellectual participation in the propaedeutic and/or therapeutic conduct of the studied cases; approval of the final version of the manuscript.

Rafael Brandão Varella: Effective participation in research orientation; intellectual participation in the propaedeutic and/or therapeutic conduct of the studied cases; approval of the final version of the manuscript.

Mayra Carrijo Rochael; Effective participation in research orientation; intellectual participation in the propaedeutic and/or therapeutic conduct of the studied cases; approval of the final version of the manuscript.

Luciana Pantaleão: Effective participation in research orientation; intellectual participation in the propaedeutic and/or therapeutic conduct of the studied cases; approval of the final version of the manuscript.

## Conflicts of interest

None declared.
